# Development and implementation of “handshake rounds”: An antibiotic stewardship intervention for hospitalized adult patients with hematologic malignancies

**DOI:** 10.1017/ash.2023.125

**Published:** 2023-04-17

**Authors:** Chelsea A. Gorsline, Ryan M. Miller, Laura J. Bobbitt, Gowri Satyanarayana, Muhamed Baljevic, Milner B. O. Staub

**Affiliations:** 1 Division of Infectious Diseases, Department of Medicine, Vanderbilt University Medical Center, Nashville, Tennessee; 2 Division of Infectious Diseases, Department of Medicine, University of Kansas Medical Center, Kansas City, Kansas; 3 Department of Pharmaceutical Services, Vanderbilt University Medical Center, Nashville, Tennessee; 4 Northside Hospital, Atlanta, Georgia; 5 Division of Hematology and Oncology, Department of Medicine, Vanderbilt University Medical Center, Nashville, Tennessee

## Abstract

**Objective:**

To design and implement “handshake rounds” as an antibiotic stewardship intervention to reduce inpatient intravenous (IV) antibiotic use in patients with hematologic malignancies.

**Design:**

Quasi-experimental analysis of antibiotic use (AU) and secondary outcomes before and and after handshake rounds were implemented.

**Setting:**

Quaternary-care, academic medical center.

**Patients:**

Hospitalized adults with hematologic malignancies receiving IV antibiotics.

**Methods:**

We performed a retrospective review of a preintervention cohort prior to the intervention. A multidisciplinary team developed criteria for de-escalation of antibiotics, logistics of handshake rounds, and outcome metrics. Eligible patients were discussed during scheduled handshake rounds between a hematology–oncology pharmacist and transplant–infectious diseases (TID) physician. Prospective data were collected over 30 days in the postintervention cohort. Due to small sample size, 2:1 matching was used to compare pre- to and postintervention AU. Total AU in days of therapy per 1,000 patient days (DOT/1,000 PD) was reported. Mean AU per patient was analyzed using Wilcoxon rank-sum test. A descriptive analysis of secondary outcomes of pre- and postintervention cohorts was performed.

**Results:**

Total AU was substantially lower after the intervention, with 517 DOT/1,000 PD compared to 865 DOT/1,000 PD before the intervention. There was no statistically significant difference in the mean AU per patient between the 2 cohorts. There was a lower rate of 30-day mortality in the postintervention cohort and rates of ICU admissions were similar.

**Conclusions:**

Conducting handshake rounds is a safe and effective way to implement an antibiotic stewardship intervention among high-risk patient population such as those with hematologic malignancies.

Antibiotic stewardship interventions are lacking among high-risk populations such as patients with hematologic malignancies. With rising rates of antimicrobial resistance and knowledge that colonization and infections with multidrug-resistant organisms (MDROs) are associated with worse outcomes in those with hematologic malignancies,^1–5^ novel approaches to stewardship in these populations are needed. Despite previous studies confirming the safety of early antibiotic de-escalation in patients with febrile neutropenia,^6–9^ the practice is sporadically implemented across North American institutions. At our center, the hematology–oncology unit in the adult hospital was the highest per-patient user of intravenous (IV) antibiotics between 2018 and 2020. An algorithm for escalation of antimicrobials for the management of febrile neutropenia was widely used; however, guidance for de-escalation of IV antibiotics to oral prophylaxis released in January 2020 was not widely adopted. In a previously published study,^10^ we reviewed findings of an internal survey of providers of high-risk febrile neutropenia patients to understand barriers to early antibiotic de-escalation in this setting. Using hypothetical clinical scenarios, we found that hematology–oncology fellows and hematology–oncology pharmacists were the groups most comfortable with early de-escalation practices. Clinical decision-making factors that drove antibiotic use (AU) were mainly related to fear, including fear of poor outcomes, severity and complexity of illness, and development of MDROs. In addition, we identified that providers desired in-person “handshake rounds” with an infectious diseases (ID) specialist to support de-escalating antibiotics. This form of stewardship was previously described by Parker et al^11^ as a successful way to reduce AU by providing face-to-face, personalized audit-and-feedback of antibiotic prescriptions. However, little has been published on this intervention,^12–16^ and to our knowledge it has not been reported with use in a high-risk population such as patients with hematologic malignancies.

Herein, we describe the development and implementation of handshake rounds as an antibiotic stewardship intervention to reduce use of IV antibiotics among patients hospitalized with hematologic malignancies. We present our comparison of AU and secondary outcomes to a preintervention cohort and a review of feedback from the hematology–oncology unit regarding the intervention. We then discuss strategies for future initiatives.

## Methods

### Preintervention planning

Multiple meetings with key stakeholders from the hematology–oncology unit, the infectious diseases division, and the antimicrobial stewardship program (ASP) at Vanderbilt University Medical Center were held to design the protocol. From these meetings, the inpatient malignant hematology teaching service was selected as the most appropriate setting for this intervention. This team is staffed by 3 medical residents, 1 hematology–oncology fellow, 1 hematology–oncology pharmacist, and 1 hematology–oncology attending physician. The team cares for patients with malignant hematological conditions, such as leukemia, lymphoma, and multiple myeloma, and is limited to simultaneous care of 16 patients. This team was selected because it includes hematology–oncology pharmacists and fellows; both were previously identified as more likely to feel comfortable with antimicrobial de-escalation.^10^ In addition, it was determined that handshake rounds should be conducted between the hematology–oncology team pharmacist and a member of the ASP team who specialized in transplant–immunocompromised host infectious diseases (TID) to ensure proper fund of knowledge. Handshake rounds between the hematology–oncology team pharmacist and a TID physician were to be conducted 2–3 times per week over 16 weeks.

### Protocol design

The previously developed internal antibiotic de-escalation protocol was reviewed, simplified for clarity, and adapted as inclusion criteria for the study. Patients included were those admitted to the teaching team, prescribed IV antibiotics and (1) afebrile for at least 48 hours, (2) hemodynamically stable, (3) had a negative work-up for infections or no infection identified, and (4) if absolute neutrophil count (ANC) <500 cells/µL, they were able to be evaluated until neutrophil recovery. Patients excluded from the study were those whom the ID consult service was managing and those in process of transfer in or out of an intensive care unit (ICU). Our initial inclusion criteria were for only those patients with ANC <500 cells/µL; however, on the first day of the study it was evident that all patients would benefit from audit and feedback, regardless of ANC. In addition, it was apparent that the definition of de-escalation was broader than envisioned during preintervention planning. For instance, many patients were prescribed 2 or 3 IV antibiotics with opportunity for de-escalating unnecessary agents such as vancomycin. Therefore, we modified our inclusion criteria to remove restriction on ANC and expand our definition of de-escalation. Cefepime, piperacillin-tazobactam, and vancomycin are the 3 most used IV antibiotics at our facility and therefore were included for analysis. Conversely, carbapenems, daptomycin, and linezolid are restricted, with low rates of use and thus were excluded from the analysis.

A clinical support tool was built in the electronic medical record to automatically populate patients on the inpatient team and visually flag those on antibiotics. A digital flyer detailing the intervention was created and distributed to oncoming members of the inpatient team prior to starting service. Deidentified postintervention data were collected on patients for whom AU feedback was provided during handshake rounds. Prospective deidentified data were collected over 30 days and stored using Excel version 16.66.1 software (Microsoft Redmond, WA).

### Preintervention cohort

Retrospective deidentified data were collected on adult patients admitted to Vanderbilt University Medical Center between January 1, 2018, to December 31, 2019, prior to release of our de-escalation protocol, to use for comparison to the postintervention cohort. Inclusion criteria were those patients with malignant hematologic conditions and aged >18 years. In addition, diagnosis codes for febrile neutropenia were used to identify patients treated with IV antibiotics. If a patient was admitted multiple times during the study window, only the first admission was included for review. Exclusion criteria included those who were adults but receiving care through the pediatric hospital and those with solid malignant tumors or rheumatologic conditions.

### Analysis

Given the complexity of these patients and the smaller size of the postintervention group in this pilot study, we created a matched 2:1 pre- to postintervention cohort to consider confounding factors. The matched preintervention cohort was created using the Stata command *ccmatch* with matching on sex (male, female), age group (<40, 40–54, 55–69, or >70 years), dichotomized absolute neutrophil count (<500 cells/µL, >500 cells/µL), and hematopoietic stem cell transplant status (none, allogeneic transplant, or autologous transplant) Stata MP, version 16.1 software (StataCorp, College Station, TX) was used for these analyses.

Some postintervention patients did not have initial matches in the preintervention cohort identified via Stata algorithm. For those without a match identified, potential matches were found by systematic relaxation of matching criteria. First, we allowed matching by age group within 1 stratum of the case (eg, if case listed as age group 2, then potential matches could be from group 1 or 3). If no match was found, we allowed for matching by dichotomized stem-cell transplant status (eg, if patient received an autologous transplant, a match would be allowed for allogeneic transplant). If there was >1 potential match after relaxing criteria, matches were selected by entering all potential matches into an online random number selector. Preintervention patients were matched only once.

Raw AU data for cefepime, piperacillin-tazobactam and vancomycin from each patient for the selected episode were extracted from the chart and were manually converted to days of therapy per 1,000 patients (DOT/1,000 PD) using a publicly available formula.^17^ Mean AU per patient between the pre- and postintervention cohorts was analyzed using the Wilcoxon rank-sum test for nonparametric distribution of data using Stata MP.

Descriptive analyses of secondary outcomes, including source of fever, ICU admission ICU, *Clostridioides difficile* infection, and all cause 30-day mortality were performed for both the pre- and postintervention cohorts. Additionally, written key informant interviews were conducted with the team pharmacist and one of the hematology–oncology attending physicians after the completion of the study. These questions were designed using the principles of feasibility studies outlined by Bowen et al^18^ and were used to gauge perceived efficacy and feasibility (see the Supplementary Material for a list of all questions). This study was deemed a quality improvement effort by the Institutional Review Board of Vanderbilt University Medical Center.

## Results

### Preintervention cohort

In total, 296 patients from 2018–2019 were identified by diagnosis code for neutropenic fever and underlying hematological disease. Retrospective chart review for demographics and patient characteristics was performed on all 296 patients. Of the 296 patients, 52 were matched in 2:1 fashion with the 26 patients in the postintervention cohort. Three patients in the postintervention cohort were unable to be matched in the first round of matching and required subsequent rounds of processing, as outlined in the methods section, to find a suitable match. Demographics and characteristics of the preintervention matched pairs are reported in Table 1.

**Table 1.** Characteristics of Pre- and Postintervention Cohorts

Characteristic	Preintervention Group (N = 52), No. (%)	Postintervention Group (N = 26), No. (%)
**Age, y**		
Range	25–82	20–86
Mean	56	58
Median	59	62.5
**Sex**		
Female	26 (50)	14 (54)
Male	26 (50)	12 (46)
**Underlying disease**		
Acute lymphoblastic leukemia	8 (15)	2 (8)
Acute myeloid leukemia	16 (31)	12 (46)
Chronic myeloid leukemia	0	1 (4)
Lymphoma	12 (23)	4 (15)
Multiple myeloma	11 (21)	5 (19)
Myelodysplastic syndrome	5 (10)	2 (8)
Relapse or progression of disease	16 (30)	9 (34)
**History of BMT**		
Autologous BMT	6 (12)	4 (15)
Allogeneic BMT	2 (4)	0
Absolute neutrophil count at start of intravenous antibiotics <500 cells/µL	26 (50)	13 (50)
Source of fever or infection identified	31 (60)	13 (50)
Admission to ICU	15 (29)	8 (30)
Admission to ICU for septic shock	2 (4)	4 (15)
*Clostridioides difficile* infection	3 (6)	3 (12)
Death within 30 d	9 (17)	3 (12)
**Length of stay, d**		
Range	2–109	3–63
Mean	14	23
Median	7	23

Note. BMT, bone marrow transplant; ICU, intensive care unit.

### Handshake rounds

Scheduled handshake rounds occurred from December 1, 2021, to March 31, 2022, 2–3 times per week with each session lasting ∼20–30 minutes. Sessions were conducted in person on the malignant hematology inpatient unit between the team’s hematology–oncology pharmacist and TID physician. Prior to each session, the TID physician spent 10–15 minutes reviewing antibiotic orders to identify potential candidates for de-escalation. Sessions were directed by the TID physician beginning with discussion of de-escalation candidates, followed by open discussion on ID or oncology topics. Handshake rounds did not occur in 3 of the weeks due to schedule conflicts and disruptions related to COVID-19. Meetings occurred in the afternoon after the inpatient team made rounds and the pharmacist presented recommendations the same day or the following morning during team rounds. Recommendations for de-escalation varied but included stopping an unnecessary agent, de-escalating from broad-spectrum IV antibiotic to narrower-spectrum IV antibiotic or de-escalating from IV antibiotic to oral antibiotic. Additional diagnostic testing, such as screening for methicillin-resistant *Staphylococcus aureus* carriage as an aid to stop vancomycin and consultation with ID, were also recommended during the study window but were not tracked.

### Antibiotic use

Total AU for cefepime, piperacillin-tazobactam, IV vancomycin, and all 3 drugs were calculated using DOT/1,000 PD and showed substantially lower absolute rates of AU in the postintervention cohort. Mean AU per patient between the pre- and postintervention cohorts was compared using Wilcoxon rank-sum test and showed similar AU rates for all antibiotics combined, slightly lower AU rates of cefepime and piperacillin-tazobactam, and higher AU rates of vancomycin. None of these differences were statistically significant. AU data are presented in Table 2.

**Table 2.** Days of Antibiotic Therapy Compared Between Pre- and Postintervention Cohorts in 2:1 Matching

Antibiotic Use	Preintervention, Days of Therapy/1,000 Patient Days	Postintervention, Days of Therapy/1,000 Patient Days	*P* Value	*Z* Value
**Total antibiotic use**				
Cefepime	508	288	…	…
Piperacillin-tazobactam	124	43	…	…
Vancomycin	232	185	…	…
Cefepime, piperacillin/tazobactam and vancomycin	865	517	…	…
**Mean antibiotic use (±SD)**				
Cefepime	7.0 (7.5)	6.7 (5.6)	.62	−0.51
Piperacillin-tazobactam	1.7 (4.9)	1 (2.3)	.98	−0.10
Vancomycin	3.2 (4.5)	4.3 (4.1)	.11	−1.61
Cefepime, piperacillin/tazobactam and vancomycin	11.9 (14.5)	11.9 (8.0)	.11	−1.61

Note. SD, standard deviation.

### Postintervention cohort secondary outcomes

Active antibiotic orders were audited for all patients admitted to the study team on days that handshake rounds occurred. Feedback was provided on 26 patients during the pilot session of handshake rounds. Characteristics of the 26 patients are presented in Table 1.

Among 26 patients, 21 (80%) had at least 1 IV antibiotic de-escalated. The remaining 5 patients were continued on IV antibiotic(s), of whom 4 had ANC < 500 cells/µL. Of these 5 patients, 3 had known infections with neutropenic fever (ANC < 500 cells/µL) that required IV antibiotic(s). Also, 2 of these patients, 1 with ANC < 500 cells/µL and 1 with ANC > 500 cells/µL, had no source of fever and/or infection identified. The patient with ANC > 500 cells/µL and no source identified was later admitted to ICU for atrial fibrillation and ultimately died due to relapsed disease.

Recurrent fevers occurred after de-escalation in 6 patients, equally among those with ANC < 500 cells/µL and ANC > 500 cells/µL. Empiric IV antibiotics were restarted in those patients who had been de-escalated. Of those 6 patients who had recurrent fever, the first episode of fever tended to have no known cause (n = 5 of 6), whereas later episodes of fever were more likely to have an infection identified (n = 5 of 6). Infections identified during recurrent fever episodes included fungal pneumonia, bacteremia, and abdominal infections. A patient with ANC > 500 cells/µL and recurrent fevers died in the setting of multiple noninfectious and infectious comorbidities.

*Clostridioides difficile* was documented in 3 patients, and of those, 2 had ANC > 500 cells/µL, and finding *C. difficile* prompted de-escalation of IV antibiotics. The other patient with *C. difficile* had neutropenic fever (ANC < 500 cells/µL) due to polymicrobial bacteremia and developed *C. difficile* while on necessary IV antibiotics.

In total, 4 patients were admitted to the ICU for septic shock, but none of these patients died. Of the 4 patients, 1 had ANC < 500 cells/µL at time of transfer to ICU and the others had ANC > 500 cells/µL. Of these 4 patients, 3 had septic shock due to bacteremia and the other patient had colitis. Also, 2 of the ICU admissions occurred after de-escalation of IV antibiotics; both had ANC > 500 cells/µL and bacteremia in setting of mucositis or colitis. These patients had bacteremia with organisms that was not treated by the empiric IV antibiotic used prior to de-escalation. The other 2 patients were de-escalated after they were transferred from the ICU.

Furthermore, 1 other patient in the postintervention cohort died of frailty and relapsed disease after IV antibiotics were de-escalated. However, antibiotics were appropriately de-escalated after work-up for neutropenic fever was unrevealing and fever did not recur after IV antibiotics were stopped. In addition, all 3 patients who died in the postintervention cohort were aged 74 or above with frailty and relapsed disease prompting transition to comfort-care measures.

### Key informant interviews

Following completion of the study, written interviews were conducted with the team hematology–oncology pharmacist and one of the hematology–oncology physicians who attended the service during the study window. Both reported a change in prescribing culture and high levels of satisfaction due to practicality of the intervention. The pharmacist denied interference with job duties, found the intervention identified issues missed on teams rounds, and expanded their ID knowledge. The pharmacist thought this intervention was successful due to their personal interest in infections and working with a TID physician subspecialty trained in immunocompromised hosts. Conversely, the pharmacist noted that an imbalance of priorities would make handshake rounds difficult. The hematology–oncology attending physician reported more comfort with de-escalation and thought the intervention would lead to long-lasting changes in prescribing habits. The attending physician was hopeful that reduced AU would lead to fewer MDROs and wanted the intervention to be continued permanently. There were no perceived negative effects from the study reported by either the pharmacist or the attending physician.

## Discussion

Antimicrobial stewardship interventions for special patient populations can be challenging to implement due to high levels of fear and anxiety among providers about patient outcomes.^10^ Here, we describe the successful implementation of handshake rounds to change antibiotic prescribing habits for patients with hematologic malignancies. Although the initial intent of the intervention was to encourage early de-escalation of IV antibiotics in the management of febrile neutropenia, it was immediately apparent that those with ANC > 500 cells/µL were receiving more IV antibiotics than needed. By expanding inclusion criteria to include those with ANC > 500 cells/µL, hematology–oncology providers became more comfortable with de-escalating antibiotics, and this positively reinforced the practice in the management of febrile neutropenia. Scheduled handshake rounds were highly effective, with de-escalation of IV antibiotics in 80% of patients in the postintervention cohort. In addition, when assessing total AU by standardized DOT/1,000 PD, AU was substantially lower among the postintervention cohort, with 517 DOT/1,000 PD compared to 865 DOT/1,000 PD in the preintervention cohort. We were unable to show a significant difference in mean AU per patient, but we suspect this was due to variation in length of stay between the 2 groups and the small sample size.

The 2 cohorts had overall similar rates of ICU admission, although the postintervention cohort did have higher frequency of ICU admissions for septic shock. However, on further analysis, ICU admissions in the postintervention cohort did not appear to be related to antibiotic de-escalation. The postintervention cohort had a lower rate of mortality at 12% compared to 17% in the preintervention cohort. In addition, mortality appeared to be related to frailty and comorbidities, such as progression or relapse of underlying disease, rather than antibiotic de-escalation. This finding is reassuring that handshake rounds can be safely and effectively implemented for these complex patients without risk of negative impacts.

Thorough preintervention planning to understand the reasons why providers were hesitant to de-escalate IV antibiotics in this population led to a desire for handshake rounds.^10^ Thoughtful development of this multidisciplinary approach strengthened the relationship between hematology–oncology providers, TID specialists, and pharmacists, and it laid a foundation of trust with mutual desire for good patient outcomes. Although not used for this study, use of a structured framework based on principles of behavior analysis, such as the behavior change wheel or the theoretical domains framework,^19,20^ may aid in understanding barriers to behavioral change. In turn, this understanding may lead to the development of interventions that avoid incorrect assumptions about what needs to change.^21–23^ In addition, interventions utilizing a multidisciplinary approach should be strongly considered; multidisciplinary interventions are supported by the Infectious Diseases Society of America recommendations for building an ASP^24^ and the literature.^25–29^ Given the success of this intervention, a multidisciplinary approach with careful preintervention planning with consideration of behaviors should be used for future interventions in high-risk populations.

This study had several limitations. The small size of the postintervention cohort was most likely responsible for the inability to demonstrate a statistically significant decrease in antibiotic use. However, our goal was to assess feasibility, safety, and potential impact of such an intervention in this patient population, which was accomplished. Because this study was designed as a quality improvement intervention, the desire for flexibility in adjusting protocols and developing a “real-world” intervention also led to the inability to fully account for bias and confounders in outcome analysis. However, by using a matched cohort, we did consider some of the major patient factors that would affect outcomes.

In conclusion, handshake rounds are a safe, effective way to shape the behavioral change needed to reduce AU, even when performed on a periodic basis and amongst high-risk, complex patients. Following the success of this intervention, our goal is to further expand handshake rounds in our autologous and allogeneic stem-cell transplant recipients.

## Supplementary material

For supplementary material accompanying this paper visit http://doi.org/10.1017/ash.2023.125. click here to view supplementary material
