# Supplementary material for: Development and implementation of “handshake rounds”: An antibiotic stewardship intervention for hospitalized adult patients with hematologic malignancies
Source: Antimicrob Steward Healthc Epidemiol. 2023 Apr 17;3(1):e76. doi: 10.1017/ash.2023.125 (PMC10127239; doi:10.1017/ash.2023.125)
Supplement: Supplementary file 1 [file S2732494X23001250sup001.docx]

**Post-Intervention Questions for Hematology/Oncology Pharmacist**

1. Acceptability/Efficacy
   1. How satisfied were you with this form of handshake stewardship (ie “pilot”)?
   2. How did this pilot fit within the hematology/oncology divisional culture?
   3. What are some perceived positive effects of this pilot?
   4. What are some perceived negative effects of this pilot?
2. Demand
   1. Was there demand for these services prior to implementation?
   2. Is there demand for making this a permanent program?
3. Implementation
   1. What aspects of the implementation of this pilot were successful?
   2. Was this pilot efficient or were there barriers that led to inefficiency?
4. Practicality
   1. To what degree were you able to accommodate the time needed for this pilot?
   2. Did this interfere with your typical job duties?
5. Adaptation/Expansion
   1. Would this pilot be adaptable for other target populations – ie, could this be applied to other inpatient teams? ​
   2. If so, what perceived benefits to the would there be to expanding?
6. Integration
   1. Do you think transitioning this pilot into a permanent program would be sustainable within the existing system?
   2. What barriers exist to making this a permanent program?

**Questions for Hematology/Oncology Attending**

1. Were you aware there was a pilot program to support de-escalation of antibiotics on patients admitted to the inpatient teaching service during the months of December 2021 to March 2022?
   - Yes / No
2. How satisfied were you with the added support for de-escalation of antibiotics in patients admitted to the inpatient teaching service?
   - Very unsatisfied / Unsatisfied / Satisfied / Very satisfied
3. Since the start of this pilot, has your level of comfort with de-escalation of antibiotics changed?
   - Less comfortable / No change / More comfortable / Significantly more comfortable
4. Do you think this pilot will lead to significant, long-lasting change in the way you prescribe antibiotics?
   - Yes / No
5. Would you like to have this pilot program continued in a permanent form?
   - Yes / No
6. Do you want this pilot program to be extended to the other inpatient hematology/oncology teams?
   - Yes / No
7. What are some of your perceived positive effects of this pilot?
   - __________________________________________
8. What are some of your perceived negative effects of this pilot?
   - __________________________________________
9. Any other thoughts or feedback on this pilot?
   - __________________________________________
